# Preoperative MEG reveals differential brain network characteristics in drug-resistant epilepsy patients based on vagus nerve stimulation response

**DOI:** 10.1007/s10072-025-08682-x

**Published:** 2026-01-12

**Authors:** Lingling Yang, Minghao Li, Hongxing Liu, Ying fan Wang, Jing Lu, Yuejun Li, Fangqing Chen, Haitao Zhu, Haiyan Ma, Yiqing Yang, Qiqi Chen, Lu Yang, Xuefeng Qu, Rui Zhang, Xiaoshan Wang

**Affiliations:** 1 Department of Neurology, Affiliated Brain Hospital of Nanjing Medical University, Nanjing, 210029 Jiangsu China; 2 Department of Neurology, The Yancheng School of Clinical Medicine of Nanjing Medical University, Affiliated Hospital 6 of Nantong University, Third People’s Hospital, Yancheng, Jiangsu 224008 China; 3 Department of Functional Neurosurgery, Affiliated Brain Hospital of Nanjing Medical University, Nanjing, 210029 Jiangsu China; 4 Department of Magnetoencephalography, Affiliated Brain Hospital of Nanjing Medical University, Nanjing, 210029 Jiangsu China

**Keywords:** Magnetoencephalography, Drug-resistant epilepsy, Functional connectivity, Vagus nerve stimulation, Network-based statistical

## Abstract

**Purpose:**

This study investigates the potential of preoperative MEG functional connectivity networks to predict the efficacy of vagus nerve stimulation (VNS) in patients with drug-resistant epilepsy (DRE).

**Methods:**

A total of 18 DRE patients and 18 healthy controls were enrolled. Resting-state MEG data were collected preoperatively, and brain network connectivity was assessed across seven frequency bands (δ, θ, α, β, γ, ripple, and fast ripple) using corrected amplitude envelope correlation (AEC-c). Network-based statistics (NBS) were employed to identify differences in connectivity patterns.

**Results:**

Compared to healthy controls, DRE patients, particularly non-responders (NR-VNS), exhibited widespread abnormal functional connectivity, including significant increases in low-frequency bands and mixed alterations in mid-to-high frequency bands. Responders (R-VNS) showed marked normalization of brain connectivity, with reductions in differences from controls, especially within alpha and beta bands. These connectivity patterns were significantly associated with treatment outcomes, indicating their potential as predictive biomarkers.

**Conclusions:**

Preoperative brain network patterns derived from multi-frequency MEG, particularly in alpha and beta bands, hold promise for predicting VNS treatment response in DRE patients. The “health status” of the brain’s network prior to implantation appears to be a crucial factor influencing therapeutic efficacy.

**Supplementary Information:**

The online version contains supplementary material available at 10.1007/s10072-025-08682-x.

## Introduction

Epilepsy is a recurrent neurological disorder characterized by sudden abnormal neuronal discharge, affecting approximately 70 million people globally [1]. Despite adequate trials of two well-tolerated and judiciously chosen antiepileptic drug (AED) regimens (including both monotherapy and combination therapy), approximately one-third of epilepsy patients remain uncontrolled, failing to achieve sustained seizure freedom [2, 3].

Vagus nerve stimulation (VNS) is a therapeutic option for patients with drug-resistant epilepsy (DRE) [4], with its efficacy gradually improving over time [5]. During follow-up periods ranging from 3 to 36 months, VNS resulted in an average reduction of 34.7% in seizure frequency (95% CI: −5.1 to 74.5) [6]. It is imperative to identify VNS responders among patients with DRE. Recent neuroimaging studies, utilizing electroencephalographic (EEG) and functional magnetic resonance image (f-MRI), suggest that VNS responders and non-responders differ in their underlying brain network organization [7–9]. Clifford et al. [10]suggest that pre-VNS implantation neurophysiological network measurements may identify VNS responders(R-VNS) and VNS non-responders (NR-VNS), with this predictive capability maintained post-implantation. A predictive model developed by Cheng et al. [11], which utilized pre-operative clinical and EEG features, distinguished R-VNS with an accuracy of 81.5% and a precision of 80.1%. This provides further evidence for the feasibility of predicting VNS response using pre-operative brain network characteristics.

Magnetoencephalography (MEG) records brain electrical activity as magnetic fields. Unlike EEG, MEG is not attenuated or distorted by the conductivity of intervening tissues, such as cerebrospinal fluid, cortical atrophy, bone, or skin. This inherent advantage renders MEG particularly valuable for pre-operative evaluation. For instance, resting-state MEG (rs-MEG) connectivity analysis has proven instrumental in identifying the seizure onset zone (SOZ), investigating alterations in pre-seizure brain connectivity patterns, and correlating changes in brain connectivity with epilepsy duration and severity [12–15], and serves as an effective tool for characterizing brain networks in epilepsy [16]. A prior investigation focused on MEG data in the 4–30 Hz band, employing network topology analysis to predict the efficacy of VNS in terms of seizure control [17]. However, MEG data in frequency bands above 30 Hz have largely remained unanalyzed. To address this, we acquired interictal rs-MEG data from pre-VNS patients and performed multi-band analysis using network-based statistical methods to predict VNS seizure outcomes.

## Methods

### Participants

This retrospective study included 18 DRE patients who underwent VNS implantation (PINS Model G111, made in Beijing, China) at Nanjing Brain Hospital’s Epilepsy Center from July 2020 to October 2023. All patients underwent thorough preoperative assessments, which included an analysis of their medical history and auxiliary tests such as 24-hour EEG, PET-CT, epilepsy protocol MRI, and MEG. From the time of VNS implantation, the epilepsy team conducted follow-up for each patient every three months—via telephone, WeChat, or outpatient visits—for at least 12 months, recording postoperative seizure frequency and assessing clinical efficacy 12 months after VNS activation. In the assessment, compared with the pre-implantation baseline value over the three months preceding implantation, a seizure frequency reduction of ≥ 50% was defined as a responder, whereas a reduction of < 50% was classified as a non-responder [9, 11, 17, 18]. Inclusion criteria for the study were as follows: (1) meeting the diagnostic criteria for DRE [2]. (2) Absence of other severe neurological or psychiatric conditions that could confound the study results or impact the patient’s ability to participate in the research. (3) Good treatment adherence. Exclusion criteria included: (1) Lesions identified on MRI are suitable for surgical excision. (2) Patients who underwent any surgical procedures or alternative antiepileptic treatments after implantation, in addition to standard medication, were excluded. (3) A follow-up period of at least one year is required. (4) Patients who ceased VNS therapy at any point during the follow-up period were excluded. (5) Interference with MEG data acquisition. (6) Subjects lost to follow-up or with incomplete medical records. Also, 18 healthy subjects were included as the healthy controls (HC). This study was approved by the Ethics Committee at the Nanjing Medical University Affiliated Brain Hospital (2022-KY111-01). All Participants provided written informed consent prior to their involvement in the research.

### Data collection

#### MEG data collection

MEG data acquisition was conducted within a magnetically shielded room using a CTF-275 MEG system. Prior to data collection, fiducial coils were positioned in front of the participants’ ears and at the nasion to ascertain their head position. During the data collection, participants were required to maintain a supine position, remain relaxed, awake, and keep their eyes closed. Any head movement exceeding 5 millimeters from the initial position before and after data collection necessitated reacquisition of the data. The MEG sampling frequency was set at 6000 Hz, with each MEG data acquisition lasting 120 s. Each participant underwent seven sets of data acquisition, followed by three-dimensional gradient noise reduction processing. The subject’s coordinates were set using a digitizer, with the line connecting the bilateral pre-auricular points as the positive X-axis, the right side as the positive direction, the nasion pointing forward as the Y-axis, and the Z-axis perpendicular to the X-Y plane, with the upward direction as positive.

#### MRI

MRI data from the subjects were collected using a 3.0T high-field superconducting magnetic resonance scanner from Siemens, Germany. The scanning parameters were consistent with those in recently published articles [19]. Two neuroscientists independently reviewed the MRI data of all subjects and, in conjunction with their medical histories and additional diagnostic examinations, systematically classified the types of epileptic seizures and conducted a detailed analysis of the underlying causes.

### Data preprocessing

The two researchers independently analyzed the MEG data of all the subjects and excluded the parts that contained obvious noise and artifacts. Subsequently, a notch filter was applied to mitigate the contamination from power line frequencies, specifically 50 Hz and its harmonics. Ocular and cardiac events, identified from Electrooculogram (EOG) and Electrocardiogram (ECG) data, are subsequently removed from MEG data using signal-space projection (SSP). The subjects selected the standard head model provided by Brainstorm and divided it into 68 regions of interest (ROIs) using the Desikan Killiany cortical segmentation map. We extracted 10-second interictal MEG data from each patient prior to VNS surgery for functional connectivity analysis. To obtain interictal data without interictal epileptiform discharges, segments of 10 s before and after without epileptiform waves were selected. For the control healthy group, 10 s of MEG data were selected. All data segments are band-pass filtered into seven frequency bands, including delta (2–4 Hz), theta (4–8 Hz), alpha (8–12 Hz), beta (12–30 Hz), gamma (30–80 Hz), ripple (80–250 Hz), and fast-ripple (250–500 Hz) bands.

We employed Corrected Amplitude Envelope Correlation (AEC-c) to evaluate functional connectivity (FC), a method known for its strong stability and reproducibility in the study of FC networks [20, 21]. Increased AEC-c values are associated with enhanced amplitude envelope coupling between cortical regions or networks [22]. For each frequency band, the amplitude envelope, which reflects the temporal fluctuation of wave amplitude within cortical regions, was derived by applying the absolute value of the Hilbert transform to band-pass filtered cortical source signals [20]. Crucially, prior to this calculation, signal pairs were orthogonalized to effectively minimize spurious connections attributed to volume conduction and field spread [23]. We have constructed a comprehensive 68 × 68 connectivity matrix by calculating AEC-c values across whole brain regions for all subjects. The MEG data was processed using Brainstorm, an electrophysiological analysis software that is freely available under the GNU General Public License (http://neuroimage.usc.edu/brainstorm).

### Data analysis

#### Statistical analysis of basic information

The Shapiro–Wilk test was employed to evaluate the distribution of the data. Comparisons of demographic and clinical variables between the DRE and HC groups were performed using Fisher’s exact test for categorical variables and either independent samples t-tests or Mann-Whitney tests for continuous variables. Statistical software employed was SPSS version 25.0 (SPSS Inc., USA). A p-value of < 0.05 was considered to indicate statistically significant differences.

#### Analysis of FC

A Network-Based Statistical (NBS) toolbox (https://sites.google.com/site/bctnet/comparison/nbs), a powerful statistical method renowned for its ability to correct for multiple comparisons within networks and its high statistical power, was utilized for the analysis of network differences between DRE patients and HC [24, 25]. In accordance with program guidelines and relevant literature, two initial threshold values were set: *p* < 0.01 to explore more widespread network connectivity alterations, and *p* < 0.001 to identify highly significant local network components. Subsequently, a null distribution was generated through 5000 permutations, and Family-Wise Error (FWE) correction was applied to control for multiple comparisons.

## Results

### Demographic and clinical characteristics

This study included 18 patients with DRE who underwent VNS implantation and were followed up for at least one year. The patient group comprised 7 females and 11 males, with a mean age of 25.61 ± 1.692 years. Among them, 7 patients were identified as responders, yielding a response rate of approximately 39%. Detailed clinical information for these DRE patients is presented in Table 1. Eighteen healthy age-matched individuals were enrolled as the HC group, consisting of 10 females and 8 males, with a mean age of 26.72 ± 1.414 years.

**Table 1:** Clinical characteristics of the VNS responder and non-responder groups

Characteristics	*R*-VNS	NR-VNS	*P*
Age (years, mean ± SD)	26.57 ± 8.10	25.0 ± 6.87	0.665^a^
Male (n, %)	5(71%)	6(55%)	0.637^b^
Duration of epilepsy (years, mean ± SD)	13.86 ± 6.149	11.82 ± 8.256	0.375^c^
MRI Negative (n, %)	3(43%)	3(27%)	0.627^b^
Number of Antiepileptic medications (n, mean ± SD)	2.86 ± 0.900	2.82 ± 0.603	1.000^c^
Pre-VNS seizure frequency (t per month, mean ± SD)	37.17 ± 82.92	11.82 ± 10.581	0.791^c^
Post-VNS seizure frequency (t per month, mean ± SD)	8.30 ± 16.284	10.32 ± 10.070	0.151^c^
Seizure reduction rate (%, mean ± SD)	72.13 ± 17.48	15.49 ± 13.58	< 0.001^c*^

^a^p was calculated using the independent samples t-test; ^b^p was calculated using the Fisher exact test; ^c^p was calculated using the Mann-Whitney test; * p is statistically significant

Abbreviations: *n* number, *SD* standard deviation, *t* time, *R-VNS* responders to Vagus nerve stimulation, *NR-VNS* non-responders to Vagus nerve stimulation, *P* P value

### Network characteristics

**Fig. 1:**
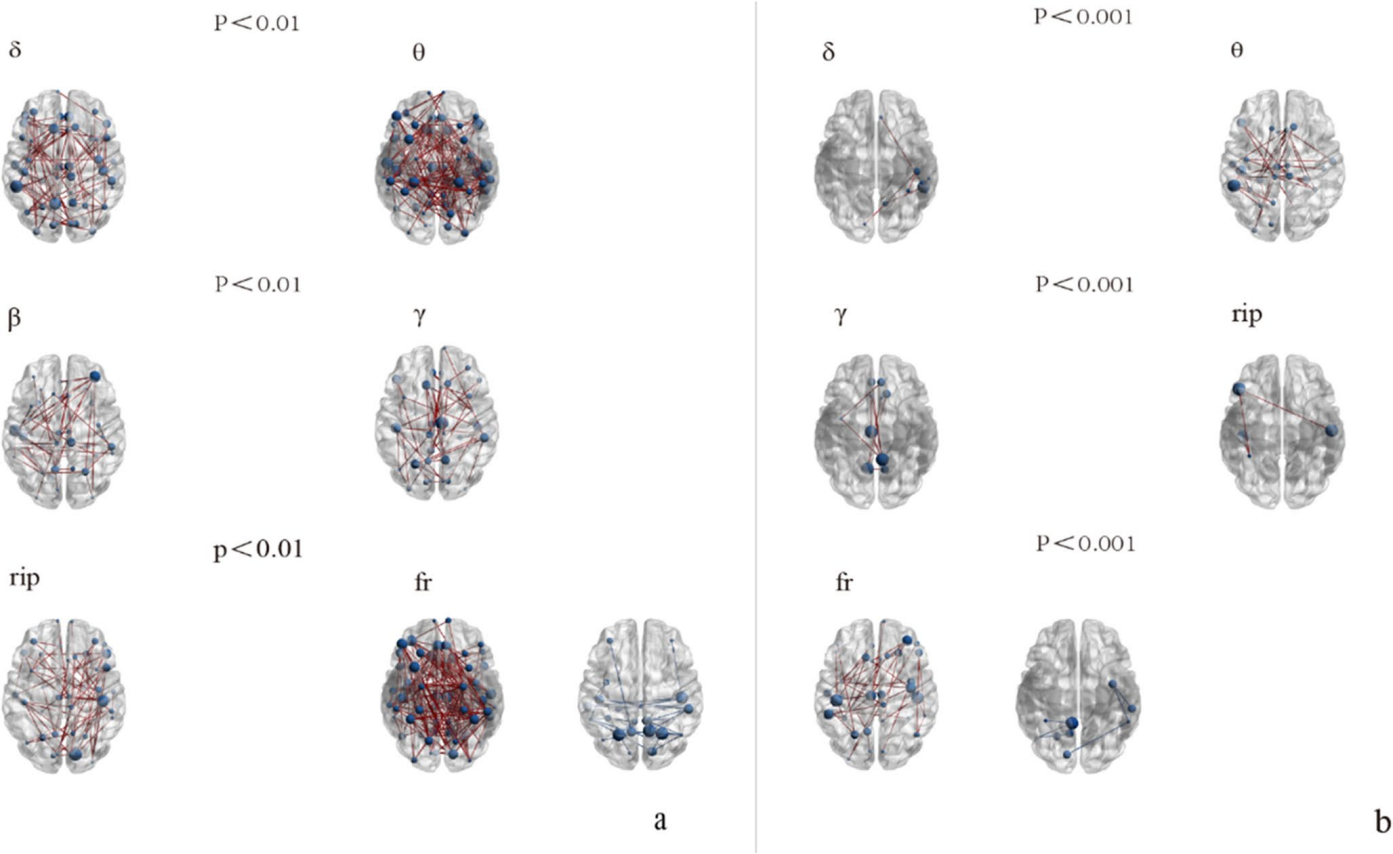
The differential brain network maps between the R-VNS group and the HC group

**Fig. 2:**
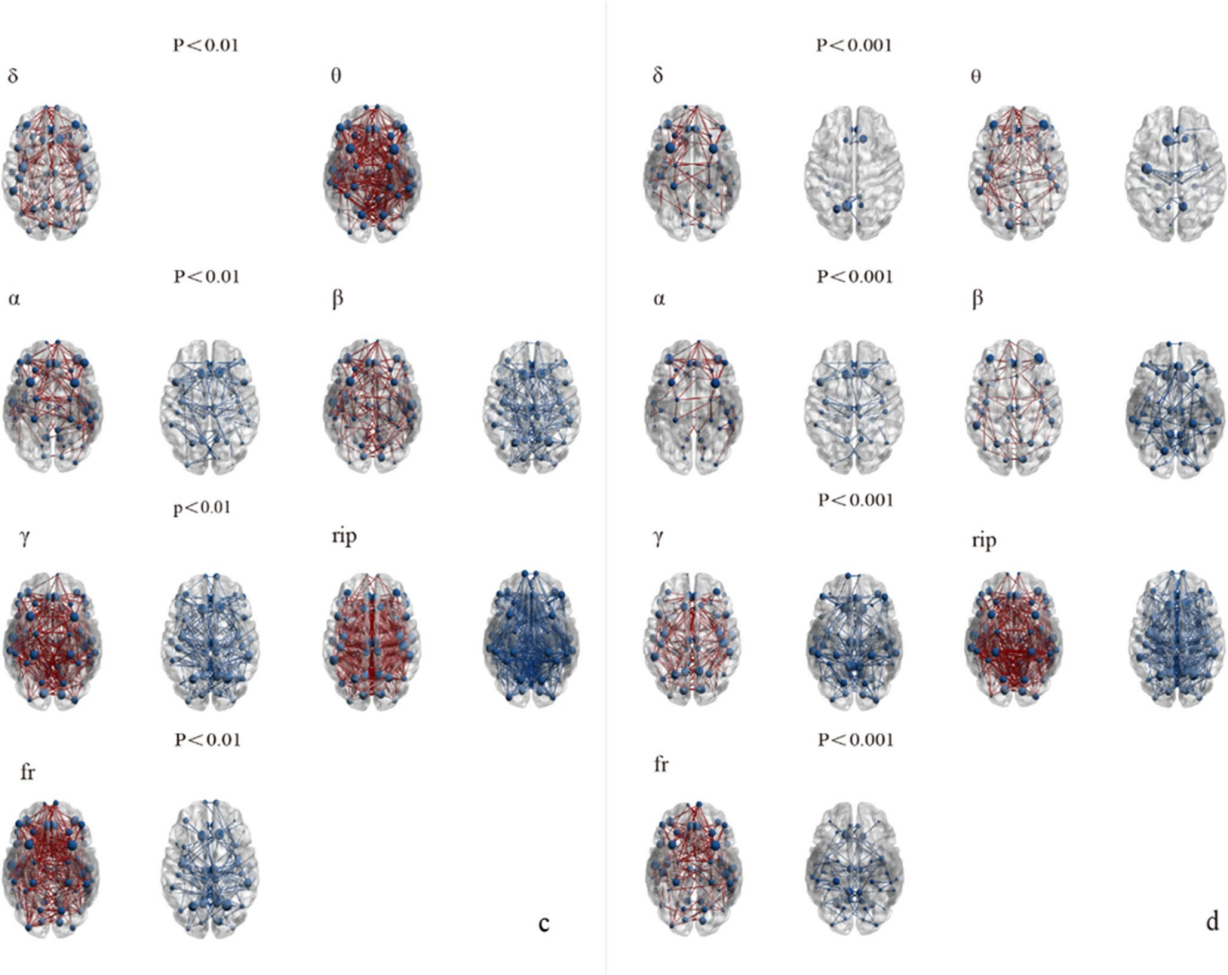
The differential brain network maps between the NR-VNS group and the HC group

**Fig. 3:**
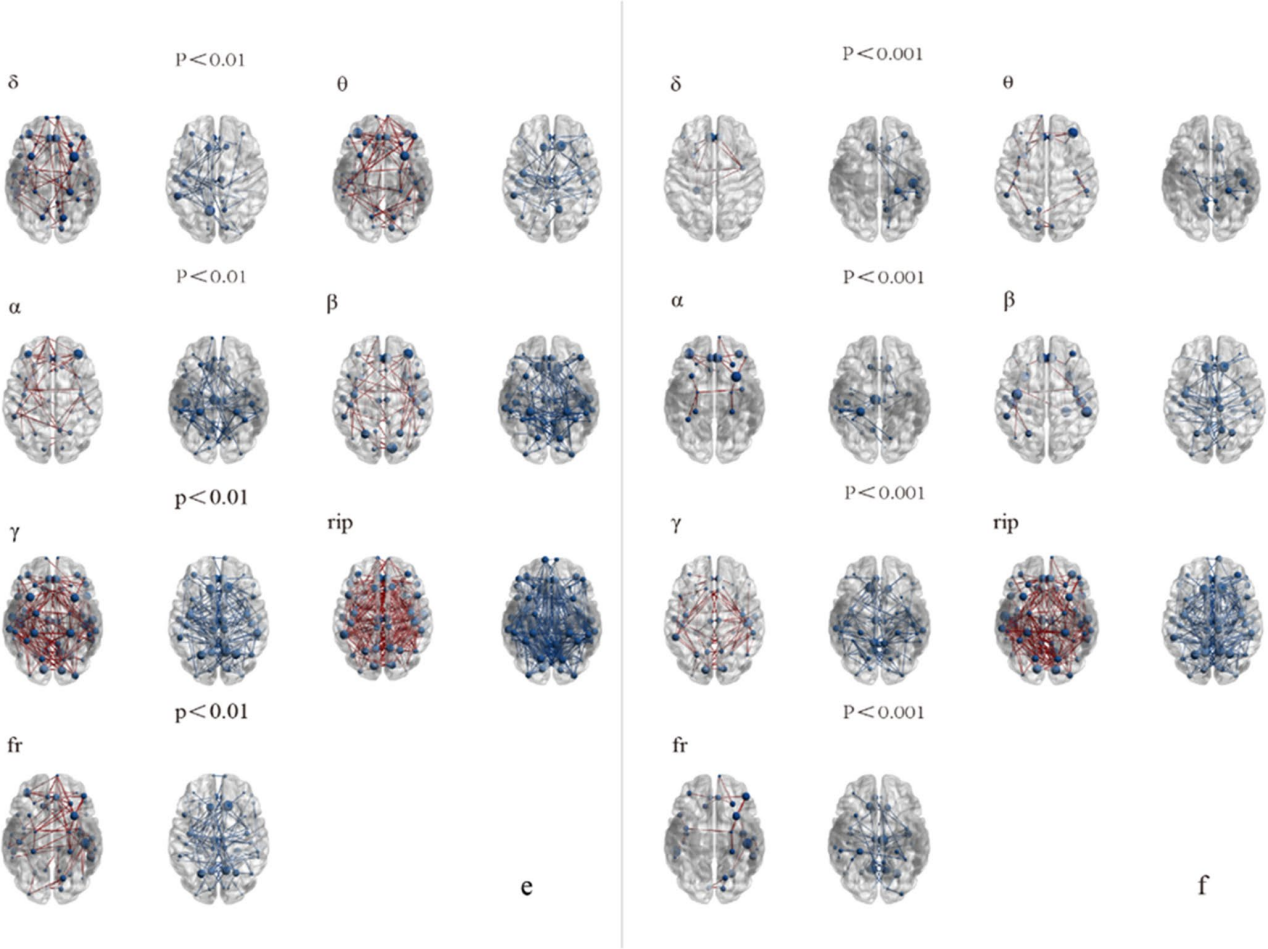
Differential brain network maps between the NR-VNS and R-VNS groups Figure 1-3 (panels a, c, e): Brain network difference maps shown at a significance threshold of *P* < 0.01 Figure 1-3 (panels b, d, f): Brain network difference maps shown at a significance threshold of *P* < 0.001 Blue circles denote brain regions showing significant differences between groups. Red lines indicate increased functional connectivity in the NR-VNS group compared to the R-VNS group, whereas blue lines represent decreased connectivity

We evaluated the changes in FC in different frequency bands (Delta, Theta, Alpha, Beta, Gamma, Ripple, Fast-ripple) between the VNS treatment groups and the healthy control group at two different threshold levels. As figure 1(a/b), Figure 2(c/d) and Figure 3(e/f).

#### Delta Band (2–4 Hz)

At a significance threshold of *P* < 0.01: *NR-VNS vs. HC*: NR-VNS patients exhibited 215 enhanced connections across 66 brain regions compared to HC. *NR-VNS vs. R-VNS*: Compared to R-VNS patients, NR-VNS patients demonstrated 75 enhanced connections in 48 brain regions and 64 decreased connections in 40 brain regions. *R-VNS vs. HC*: R-VNS patients displayed 110 enhanced connections across 60 brain regions compared to HC.

At a stricter threshold of *P* < 0.001: *NR-VNS vs. HC*: NR-VNS patients displayed 109 enhanced connections in 55 brain regions. Additionally, 17 brain regions and 17 connections showed decreased connectivity. *NR-VNS vs. R-VNS*: Compared to R-VNS, NR-VNS patients exhibited 14 enhanced connections in 10 brain regions and 19 decreased connections in 16 brain regions. *R-VNS vs. HC*: R-VNS patients showed 8 enhanced connections in 9 brain regions compared to HC.

#### Theta Band (4–8 Hz)

At a significance threshold of *P* < 0.01: *NR-VNS vs. HC*: NR-VNS patients exhibited 370 enhanced connections across 68 brain regions compared to HC. *NR-VNS vs. R-VNS*: Compared to R-VNS, NR-VNS patients demonstrated 106 enhanced connections in 57 brain regions and 77 decreased connections in 46 brain regions. *R-VNS vs. HC*: R-VNS patients displayed 170 enhanced connections across 67 brain regions compared to HC.

At a stricter threshold of *P* < 0.001: *NR-VNS vs. HC*: NR-VNS patients displayed 169 enhanced connections in 63 brain regions. Furthermore, 34 connections across 31 brain regions showed decreased connectivity. *NR-VNS vs. R-VNS*: Compared to R-VNS, NR-VNS patients exhibited 4 enhanced connections in 4 brain regions and 26 decreased connections in 23 brain regions. *R-VNS vs. HC*: R-VNS patients showed 39 enhanced connections in 28 brain regions compared to HC.

#### Alpha Band (8–12 Hz)

No statistically significant differences were observed in whole-brain functional connectivity in the alpha band between R-VNS and HC, at either *P* < 0.01 or *P* < 0.001.

At a significance threshold of *P* < 0.01: *NR-VNS vs. HC*: NR-VNS patients exhibited 161 enhanced connections in 65 brain regions and 129 decreased connections in 59 brain regions compared to HC. *NR-VNS vs. R-VNS*: Compared to R-VNS, NR-VNS patients demonstrated 71 enhanced connections in 48 brain regions and 107 decreased connections in 58 brain regions.

At a stricter threshold of *P* < 0.001: *NR-VNS vs. HC*: NR-VNS patients displayed 75 enhanced connections in 39 brain regions and 66 decreased connections in 46 brain regions compared to HC. *NR-VNS vs. R-VNS*: Compared to R-VNS, NR-VNS patients exhibited 20 enhanced connections in 15 brain regions and 31 decreased connections in 27 brain regions.

#### Beta Band (13–30 Hz)

At a significance threshold of *P* < 0.01: *NR-VNS vs. HC*: NR-VNS patients exhibited 203 enhanced connections in 68 brain regions and 201 decreased connections in 67 brain regions compared to HC. *NR-VNS vs. R-VNS*: Compared to R-VNS, NR-VNS patients demonstrated 94 enhanced connections in 59 brain regions and 155 decreased connections in 61 brain regions. *R-VNS vs. HC*: R-VNS patients displayed 55 enhanced connections in 43 brain regions compared to HC.

At a stricter threshold of *P* < 0.001: *NR-VNS vs. HC*: NR-VNS patients displayed 107 enhanced connections in 61 brain regions and 119 decreased connections in 62 brain regions compared to HC. *NR-VNS vs. R-VNS*: Compared to R-VNS, NR-VNS patients exhibited 9 enhanced connections in 10 brain regions and 82 decreased connections in 49 brain regions. *R-VNS vs. HC*: No significant changes were observed in the beta band for R-VNS patients compared to HC at this threshold.

#### Gamma Band (30–80 Hz)

At a significance threshold of *P* < 0.01: *NR-VNS vs. HC*: NR-VNS patients exhibited 359 enhanced connections in 67 brain regions and 249 decreased connections in 68 brain regions compared to HC. *NR-VNS vs. R-VNS*: Compared to R-VNS, NR-VNS patients demonstrated 202 enhanced connections in 67 brain regions and 195 decreased connections in 65 brain regions. *R-VNS vs. HC*: R-VNS patients displayed 54 enhanced connections in 42 brain regions compared to HC.

At a stricter threshold of *P* < 0.001: *NR-VNS vs. HC*: NR-VNS patients displayed 246 enhanced connections in 67 brain regions and 195 decreased connections in 64 brain regions compared to HC. *NR-VNS vs. R-VNS*: Compared to R-VNS, NR-VNS patients exhibited 102 enhanced connections in 54 brain regions and 123 decreased connections in 56 brain regions. *R-VNS vs. HC*: R-VNS patients showed 10 enhanced connections in 10 brain regions compared to HC.

#### Ripple Band (80–250 Hz)

At a significance threshold of *P* < 0.01: *NR-VNS vs. HC*: NR-VNS patients exhibited 540 enhanced connections and 532 decreased connections in 68 brain regions compared to HC. *NR-VNS vs. R-VNS*: Compared to R-VNS, NR-VNS patients demonstrated 354 enhanced connections in 68 brain regions and 372 decreased connections in 67 brain regions. *R-VNS vs. HC*: R-VNS patients displayed 60 enhanced connections in 49 brain regions compared to HC.

At a stricter threshold of *P* < 0.001: *NR-VNS vs. HC*: NR-VNS patients displayed 424 enhanced connections and 426 decreased connections in 68 brain regions compared to HC. *NR-VNS vs. R-VNS*: Compared to R-VNS, NR-VNS patients exhibited 213 enhanced connections in 66 brain regions and 266 decreased connections in 67 brain regions. *R-VNS vs. HC*: R-VNS patients showed 3 enhanced connections in 4 brain regions compared to HC.

#### Fast-ripple Band (250–500 Hz)

At a significance threshold of *P* < 0.01: *NR-VNS vs. HC*: NR-VNS patients exhibited 386 enhanced connections in 68 brain regions and 162 decreased connections in 63 brain regions compared to HC. *NR-VNS vs. R-VNS*: Compared to R-VNS, NR-VNS patients demonstrated 89 enhanced connections in 51 brain regions and 117 decreased connections in 64 brain regions. *R-VNS vs. HC*: R-VNS patients displayed 198 enhanced connections in 66 brain regions and 31 decreased connections in 26 brain regions compared to HC.

At a stricter threshold of *P* < 0.001: *NR-VNS vs. HC*: NR-VNS patients displayed 193 enhanced connections in 65 brain regions and 112 decreased connections in 55 brain regions compared to HC. *NR-VNS vs. R-VNS*: Compared to R-VNS, NR-VNS patients exhibited 4 enhanced connections in 4 brain regions and 61 decreased connections in 37 brain regions. *R-VNS vs. HC*: R-VNS patients showed 46 enhanced connections in 39 brain regions and 7 decreased connections in 9 brain regions compared to HC.

## Discussion

This study employed multi-band MEG technology and utilized the AEC-c analysis method to investigate preoperative whole-brain FC network features in patients with DRE, further elucidating the differences in connectivity between brain regions in the R-VNS and NR-VNS groups. Multiple previous studies [11, 17, 18, 26] have demonstrated that using brain FC can be a feasible and effective approach to predict the treatment response of DRE patients to VNS therapy.

The primary findings of this study highlight several key points. Consistent with previous reports [27, 28], using a relaxed statistical threshold (*P* < 0.01), both R-VNS and NR-VNS patients with DRE exhibited significant alterations in FC compared to HC. Notably, relative to the HC group, NR-VNS patients displayed more widespread and pronounced FC abnormalities, whereas the R-VNS group showed a trend toward FC normalization. Importantly, applying a more stringent significance threshold (*P* < 0.001) revealed that R-VNS patients exhibited fewer, or even no, detectable differences in FC compared to HC, especially within the Alpha and Beta frequency bands. Before VNS treatment, NR-VNS patients already exhibited severe and widespread functional connectivity abnormalities, and the highly disordered network states may serve as the neurophysiological basis for their insensitivity to VNS therapy in some patients with DRE [29]. As early as 2015, a study had already demonstrated that VNS responders exhibited lower levels of delta and alpha band synchronicity compared to non-responders [30]. This finding is consistent with our study, where R-VNS showed normalization, while NR-VNS displayed widespread connectivity abnormalities. These findings suggest that the “health status” or “degree of abnormality” of a patient’s brain functional connectivity prior to surgery—especially in the Alpha and Beta bands—can be regarded as a key biomarker for predicting their response to VNS therapy. A study [11] based on preoperative EEG signals analyzing network topology and entropy features indicates that VNS responders have more efficient alpha-band brain networks, especially in the top-parietal regions, compared to non-responders.

The inherent heterogeneity and network complexity in the etiology and localization of the epileptogenic zone among the drug-resistant epilepsy patients included in this study are the primary explanation for their poor response to conventional pharmacological therapy. The exact mechanisms underlying VNS treatment for DRE remain unclear. One of its therapeutic effects is thought to involve modulating brain network activity. In patients with DRE, synaptic remodeling disrupts structural and functional connections within neural networks, impairing information transfer. This leads to the replacement of normal neuronal synchronization by pathological synchronization, which in turn disrupts excitation-inhibition dynamics and triggers abnormal hypersynchronous neuronal discharges, ultimately resulting in recurrent epileptic seizures [31, 32]. Current evidence suggests that VNS modulates brain synchronization via the vagal afferent network, downregulating it to a less epileptogenic state, thereby exerting anti-seizure effects [33–35]. However, when the brain networks are already significantly disordered or abnormal, VNS may struggle to fully rectify these issues. Its primary role appears to be in fine-tuning or augmenting relatively normal networks rather than fundamentally repairing severely disrupted ones [8, 35].

Moreover, our findings suggest that even within this complex patient group, differences in VNS efficacy may still be influenced by factors related to the mechanism of neuromodulation (such as brain network characteristics), providing a new direction for understanding the mechanism of action of VNS and optimizing patient selection. Furthermore, we observed a phenomenon where, at one year post-VNS implantation, there was no significant difference in seizure frequency between the two groups. Even with a 50% reduction rate achieved, the absolute benefit of VNS, from the perspective of the patient’s actual seizure burden, may still require further evaluation. Even if VNS can eliminate more than half of the seizures, for some patients, the residual seizure frequency might still remain high enough that, in terms of absolute frequency, it does not significantly differ from those patients considered NR-VNS. Previous studies [9, 11, 18] have primarily focused on pediatric patients, whose brains exhibit greater neuroplasticity, potentially leading to a better therapeutic response [36]. Another possible reason for this outcome may be insufficient follow-up duration, which has not yet fully reflected the efficacy of VNS. Some studies have shown that the effects of VNS implantation gradually become evident over 18 to 24 months [4], with the benefits increasing progressively over time. However, our research prompts reconsideration of clinical decision-making and patient management, highlighting that clinicians should evaluate not only the relative reduction in postoperative seizure frequency but also the actual seizure burden and quality of life of patients.

To our knowledge, this study represents the first investigation to explore functional brain networks in patients with drug-resistant epilepsy undergoing VNS using multi-frequency band preoperative MEG integrated with NBS. NBS offers superior statistical power in controlling for family-wise error rates compared to conventional methods [24]. Moreover, the number of network differences identified using the NBS method at different thresholds was similar, indicating that variations in feature weights had minimal impact on the prediction outcomes [18]. Additionally, MEG offers high temporal and spatial resolution (spatial resolution up to 1 mm). It directly detects neural electrical activity originating from intracellular synaptic currents, unaffected by skull and scalp signal attenuation. Furthermore, muscle or eye movements during MEG monitoring do not interfere with the recordings [13]. Although scalp EEG is easy to access, its signals are often weakened and contaminated by noise from the skull and scalp [37]. In contrast, SEEG can detect activity in deep and extensive brain regions but carries risks such as bleeding, infection, and neurological deficits due to its invasive nature [38]. fMRI provides a non-invasive method to indirectly reflect neural activity and functional networks in the brain by measuring the synchronized fluctuations of blood oxygen level-dependent (BOLD) signals, but its low temporal resolution limits the ability to differentiate the precise influence of regional neuronal activation on adjacent neuronal firing [38, 39].

One limitation of this study is its limited sample size, which thereby precluded subgroup analyses based on etiology—a common challenge in VNS research [17, 18, 40–44]. Furthermore, as a retrospective study, this design may introduce recall bias, making it challenging to precisely ascertain seizure frequency. Despite this, our main focus was to investigate the preoperative brain network differences between R-VNS and NR-VNS in patients with DRE. Regularly recording the frequency of epileptic seizures can help reduce the impact of recall bias. Future studies can conduct prospective longitudinal research, enhance the awareness of epilepsy family members in recording disease information, thereby improving the quality of follow-up and obtaining more comprehensive and accurate clinical data. Additionally, by expanding the sample size and conducting subgroup analyses based on different clinical characteristics for various types of epilepsy, further confirmation of our research findings can be achieved.

## Conclusions

This study shows that preoperative resting-state MEG brain networks can predict how well patients with drug-resistant epilepsy (DRE) will respond to VNS treatment. Patients who benefit from VNS (R-VNS) have more normal brain networks, especially in the alpha and beta frequency bands, compared to non-responders (NR-VNS). This suggests that VNS works better by influencing healthier brain networks rather than fixing severely damaged ones. Therefore, analyzing a patient’s pre-surgery brain network patterns can help identify the best candidates for VNS, allowing for personalized treatment, reducing costs, and improving patient satisfaction.

## Supplementary Information

Below is the link to the electronic supplementary material.

Supplementary Material 1 (EPS 1.85 MB)

Supplementary Material 2 (DOCX 23.7 KB)

## Data Availability

The data that support the findings of this study are available on request from the corresponding author. The data are not publicly available due to privacy or ethical restrictions.
