# Supplementary material for: Preoperative MEG reveals differential brain network characteristics in drug-resistant epilepsy patients based on vagus nerve stimulation response
Source: Neurol Sci. 2026 Jan 12;47(1):143. doi: 10.1007/s10072-025-08682-x (PMC12791077; doi:10.1007/s10072-025-08682-x)
Supplement: Supplementary file 2 — Supplementary Material 2 (DOCX 23.7 KB) [file 10072_2025_8682_MOESM2_ESM.docx]

**S1: Clinical characteristics of each subject**

|  | Sex, M/F | Age, years | Duration of epilepsy, years | Type of epilepsy, Generalized/Focal/unknown | MRI, Negative/Focal/Multifocal | Number of Antiepileptic medications, n | Pre‐VNS seizure frequency, t per month | Post‐VNS seizure frequency, t per month | Seizure reduction rate, % |
| --- | --- | --- | --- | --- | --- | --- | --- | --- | --- |
| 1 | F | 29 | 24 | Focal | Negative | 4 | 7.5 | 3 | 60 |
| 2 | M | 36 | 20 | Focal | Multifocal | 2 | 8.57 | 1.5 | 82.5 |
| 3 | M | 20 | 10 | Generalized | Multifocal | 3 | 10.71 | 5 | 53.31 |
| 4 | M | 17 | 6 | Focal | Focal | 3 | 0.42 | 0.1 | 76.19 |
| 5 | M | 31 | 13 | Focal | Focal | 4 | 225 | 45 | 80 |
| 6 | M | 18 | 13 | Focal | Negative | 2 | 0.5 | 0 | 100 |
| 7 | F | 35 | 11 | Focal | Negative | 2 | 7.5 | 3.53 | 52.93 |
| 8 | M | 16 | 10 | Focal | Focal | 2 | 3 | 2.5 | 16.67 |
| 9 | F | 31 | 15 | Focal | Multifocal | 3 | 10 | 6 | 40 |
| 10 | F | 29 | 6 | Focal | Multifocal | 3 | 25 | 25 | 0 |
| 11 | F | 36 | 24 | Focal | Negative | 3 | 12 | 12 | 0 |
| 12 | M | 20 | 4 | Focal | Multifocal | 4 | 2.5 | 2 | 20 |
| 13 | M | 23 | 8 | Focal | Multifocal | 2 | 32 | 30 | 6.25 |
| 14 | F | 18 | 3 | Focal | Negative | 3 | 6.5 | 5 | 23.08 |
| 15 | F | 26 | 10 | Focal | Multifocal | 3 | 2 | 2 | 0 |
| 16 | M | 16 | 4 | Focal | Negative | 3 | 4.5 | 4 | 11.11 |
| 17 | M | 28 | 26 | Focal | Multifocal | 3 | 7.5 | 5 | 33.33 |
| 18 | M | 32 | 20 | Focal | Multifocal | 2 | 25 | 20 | 20 |

note: F, female; M, male; n, number; VNS, Vagus nerve stimulation.
